# Predictors of oral healthcare utilization and satisfaction among Indian migrants and the host population in the Netherlands

**DOI:** 10.1186/s12903-024-04988-y

**Published:** 2024-10-15

**Authors:** Amandeep Pabbla, Denise Duijster, Irene H. A. Aartman, Charles Agyemang

**Affiliations:** 1 Department of Oral Public Health, Academic Centre for Dentistry Amsterdam (ACTA), University of Amsterdam and VU University, Gustav Mahlerlaan 3004, Amsterdam, 1081 LA The Netherlands; 2 Department of Public Health, Academic Medical Centre (AMC), University of Amsterdam, Amsterdam, The Netherlands; 3 Division of Endocrinology, Diabetes, and Metabolism, Department of Medicine, Johns Hopkins University, Baltimore, Maryland USA

**Keywords:** Dental care utilization, Andersen model, Asian Indians, Immigrants, Oral health

## Abstract

**Background:**

The aim of this study was to explore predictors associated with reasons for visiting an oral healthcare professional (OHP) and satisfaction with OHPs in the Netherlands among the Indian migrants and the host population.

**Methods:**

A random sample was obtained for this cross-sectional questionnaire study. Variables were classified according to the Andersen Behavioural Model of Health Services Utilization. Multivariable binary logistic regression analysis was conducted to identify significant predictors for reasons for visiting an oral healthcare professional (OHP) (routine checkups and preventive care or visiting only for pain and/or treatment) and satisfaction with OHPs (satisfied or dissatisfied).

**Results:**

The sample consisted of 391 participants (Indian migrants = 147 and host population = 244). Indian migrants with higher internal locus of control (LoC) [OR = 7.73 (95% CI: 2.13;27.99)], more trust in OHPs [OR = 4.12 (95% CI:1.68;10.14)] and higher integration level [OR = 1.09 (95% CI:1.03;1.17)] had higher odds of visiting an OHP for routine checkups and preventive care. In the host population, having dental insurance [OR = 2.64 (95% CI:1.00;6.95)] was significantly associated with increased odds of visiting an OHP for routine checkups and preventive care. For satisfaction, Indians with low paid jobs [OR = 16.26 (95% CI:2.83;93.36)] and those with higher integration levels [OR = 1.29 (95% CI:1.16;1.42)] had higher odds of being satisfied with the Dutch OHPs. Among the host population, those with more trust in OHPs [OR = 2.86 (95% CI:1.19;6.88)] had higher odds of being satisfied.

**Conclusion:**

Our study emphasize that integration levels and trust emerged as two crucial factors, policy makers can leverage upon to improve access to care for Indian migrants.

**Clinical trial:**

N.A as this is a survey based cross sectional study.

**Supplementary Information:**

The online version contains supplementary material available at 10.1186/s12903-024-04988-y.

## Introduction

Access to oral healthcare services is a vital aspect of overall well-being, yet it remains a significant public health concern across the globe. Marginalized populations, such as migrants, often encounter various barriers to accessing healthcare in the destination country. According to the 2020 International Migration Report, the number of international migrants reached 281 million in 2020, which equates to 3.6% of the global population [1] The migration process intricately weaves together economic, social, and emotional disruptions, significantly affecting access to oral healthcare in the host country. A systematic review indicated a generally low utilization of oral health services among migrants in the host country [2]. Moreover, there are notable variations in the type of oral healthcare sought by migrants, as they tend to opt for emergency care more frequently than routine dental checkups which are more commonly observed in the host population [3–5].

These variations in access to oral healthcare are particularly pronounced among individuals migrating from low-and-middle-income countries, such as Bangladesh, Sri Lanka, Pakistan, and India. In these countries, the organization and regulatory framework of oral healthcare systems differ significantly from those in high-income countries, as highlighted by Batra et al. [6]. Barriers in accessing oral healthcare services due to lack of dental insurance coverage [7, 8] unfamiliarity with the healthcare system [9, 10] language barriers [6, 11], or financial constraints [2] can consequently hinder the migrants’ oral healthcare use. Another important influence is the difference in their cultural beliefs and practices regarding oral health from the destination country. They may prioritize oral health differently or have different attitudes towards dental care compared to the host country [6, 11–14] Migrants may also have limited knowledge or be unaware of the oral health practices, preventive care, and the oral healthcare system in the new country [11, 13, 15]. This lack of awareness can contribute to a higher prevalence of dental problems, such as dental caries and gum disease, as well as a delay in seeking treatment [7, 16].

There is a significant number of Indians migrating to different countries, including the Netherlands. As of 2019, an estimated 58,460 Indians (exclusive of Surinamese Hindustanis) are living in the Netherlands and this number has been rising annually [17]. The Indian community in the Netherlands has a diverse array of cultural traditions, norms, beliefs, and customs that are distinctly their own. Studies conducted worldwide have indicated that Indian migrants often face oral health challenges, including a high prevalence of dental caries, periodontal diseases, and missing teeth [6, 18]. Factors such as socioeconomic status, education level, and health literacy can influence the uptake of oral healthcare services, leading to unequal access and differences in utilization patterns [19, 20]. However, studies on oral healthcare utilization, especially the reasons for visiting an oral healthcare professional (OHP) and satisfaction level with oral healthcare professionals (OHPs) are almost non-existent, except a few exceptions [4, 6].

A widely recognized model for comprehending healthcare utilization, including oral healthcare among diverse populations like migrants, is the Andersen Behavioural Model of Health Services Utilization [21]. This conceptual framework provides valuable insights into the complex interplay of predisposing, enabling, and need factors that shape (oral) healthcare-seeking behaviours [21, 22]. Predisposing factors include demographic characteristics, cultural beliefs, and individual perceptions that shape an individual’s propensity to seek (oral) healthcare. Enabling factors encompass the resources, social support, and oral healthcare infrastructure available to individuals. Access to insurance coverage, income levels, language proficiency, and the presence of community support networks are critical enabling factors that can facilitate or hinder migrants’ utilization of oral) healthcare. The need factor pertains to an individual’s perceived or evaluated (oral) health needs, including oral health problems, pain, and functional limitations.

Personal views of the urgency and severity of oral health issues, as well as subjective assessments of oral health status, can significantly impact oral healthcare-seeking behaviours. Also, oral healthcare utilization is explained more comprehensively by using explanatory variables such as reasons for dental visits and satisfaction levels with oral healthcare professionals compared to visiting or not visiting a dentist [23]. Literature underscores distinctive patterns between migrants and the host population in terms of OHP visits, with migrants often either refraining from regular visits or seeking care primarily for emergency reasons, in contrast to the host population’s tendency to prioritize preventive and routine dental visits [2, 7]. Moreover, the connection between satisfaction with OHPs and levels of acculturation among migrants highlights the need for a nuanced examination of factors influencing oral healthcare-seeking behaviour [5, 7]. Therefore, this study aimed to explore the predictor variables associated with oral healthcare utilization among the Indian migrants and the host population in the Netherlands using the model of Andersen. Oral healthcare utilization is assessed using two outcomes: reasons for visiting an OHP and satisfaction with OHPs in The Netherlands.

## Methods

For this study, we followed a cross-sectional design. We gathered data on the Indian migrants and the host population via questionnaires. The Medical Ethics Review Board of the Medical Centre of the VU University Amsterdam (reference number 2020.479), as well as the Ethical Committee of the Academic Centre for Dentistry Amsterdam (ACTA) (reference number 91550) provided approval for this study.

Comparisons on oral health status, oral health behaviours and oral healthcare utilization between Indian migrants and the host population using this dataset showed that reporting oral impact on daily performances (OIDP) was higher among Indians compared to the host population. In contrast, the odds of Indians reporting gum diseases was lower than the host population. But the two groups differed in the form of sugar consumed. However, Indian migrants were less likely to visit a dental professional compared to the host population [24]. In addition, the association between integration and oral health status, behaviours and oral healthcare utilization among Indian migrants has also been published [25].

### Study population and sampling

The study population consisted of two groups, Indian migrants and the host population. The inclusion criteria for the Indian migrants were adult migrants 18 years and above, born in India and living in the Netherlands for at least five years [24]. For the host population, we included those aged 18 years and above and born and living in The Netherlands. There were no exclusion criteria. Indian migrants in the Netherlands are primarily concentrated in five major cities, Amsterdam (including Amstelveen), Utrecht, Rotterdam, The Hague, and Eindhoven (CBS), which is reflected in our study’s sample.

**Fig. 1:**
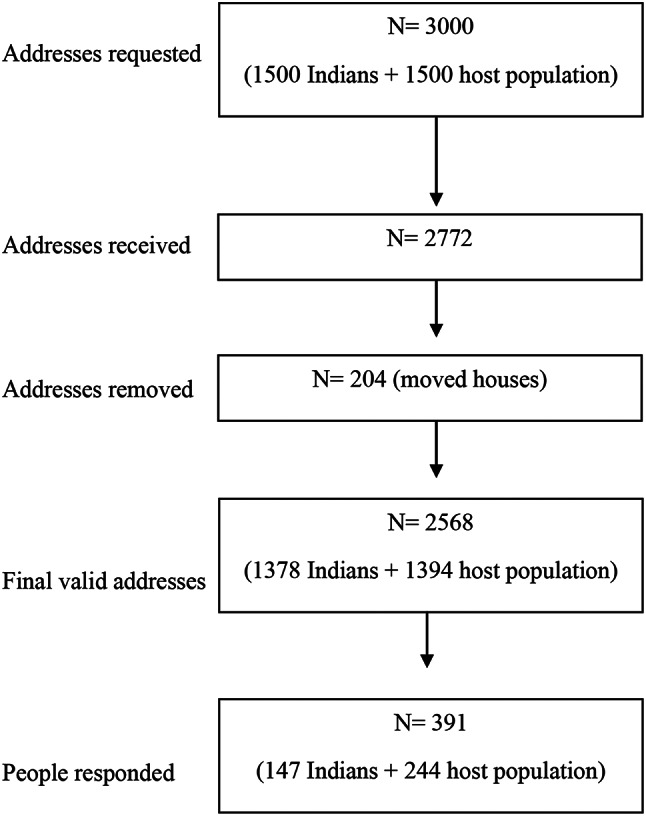
Flowchart of sample size

The Rijksdienst voor Identiteitsgegevens (RvIG) authorised the Central Bureau of Statistics, Netherlands (CBS) to draw a random stratified sample of 300 Indian migrants and 300 adult host population from the abovementioned five cities, using the registry of Dutch Municipalities (Basis Registratie Personen, BRP). This gave us a sample of 1500 Indian migrants (300 participants in each of the five cities) and 1500 adults in the host population, with a total sample size of 3000. The power calculation for this sample size was based on the aim of one of our previous studies [24]. Out of the requested sample of 3000, we got postal addresses as contact details of 1,378 Indian migrants and 1,394 adults from the host population. Out of these, 204 people had moved houses, giving us a final sample of 2,568 people (Fig. 1). The sampling was performed in January 2021.

### Data collection and processing

Since this study was a part of the larger PhD project, the details of data collection have been described in detail in our previous study [24]. Briefly, we conducted a questionnaire survey in which all participants received a survey questionnaire including a written consent form that was required to have the participant’s signature. We also included a return envelope and information letter with a link for filling in the questionnaire online/ digitally as well. The data collection was conducted between February 2021 and April 2021. Those who returned the filled questionnaire had signed the informed consent form and were included in the study.

### Variables recorded

We included two outcome variables: the reason for visiting an OHP and satisfaction with OHPs in The Netherlands. The question asked was “What is the usual reason for you to visit the dentist?”, with response options ‘consultation or advice’, ‘routine check-up’, ‘pain or trouble with the teeth’, ‘for treatment’ or ‘no visits at all’. For the analysis, we aggregated these five responses into two categories as routine checkups and preventive care (1) and no visits or visit only for pain and treatment (0). Satisfaction was asked as “How satisfied are you with the dentist?” and the responses were measured on 5-point Likert scale as completely satisfied to completely unsatisfied. These responses were also dichotomized for analysis as satisfied (completely satisfied, satisfied, neutral) and unsatisfied (unsatisfied, completely unsatisfied).

Included variables were classified according to Andersen’s model [21]. The predisposing factors were defined as individual or sociodemographic factors that exist before the onset of disease, and included age (continuous), gender (male, female), marital status (married, single), and locus of control (LoC). For the latter, we employed the Multidimensional Oral Health Locus of Control Scale (MOHLCS). This instrument assesses to what degree individuals interpret how their oral health depends on their own ability and efforts or on factors, such as the dentist, or other persons, or is simply the result of fate or chance [26]. The MOHLCS contains 26 items scored on a four-point Likert-type scale ranging from ‘strongly disagree’ to ‘strongly agree’. This scale yields four subscales reflecting various aspects of oral health LOC. Subscale scores were calculated by summing the scores of individual items within each subscale and dividing the total by the number of items. Higher scores indicated a stronger endorsement of the corresponding MOHLCS subscales. The internal LoC (range 11–44) reflects personal control, while external-dentist (range 6–24), signifies trust in dentist advice and prevention methods. Furthermore, external-chance (range 7–28) indicates belief in chance and external-socialization agents (range 2–8), signifies influence from social connections (such as family, friends, colleagues, and relatives) on oral health outcomes [26].

Enabling factors included resources that may help to successfully access oral healthcare facilities. These were income (low ≤ €2600/month, medium €2600-€4000/month, high ≥ €4000/month) [27], education (low to medium = less than graduation, high = graduation and above), occupation (paid job, unpaid), dental insurance (yes, no), and social support (scores 9–45). Social support comprised of nine items and was measured on a 5-point Likert scale. An aggregate of the total scores was taken and higher scores indicated greater levels of social support [28]. For the Indian migrants, additional variables were added as enablers. One of these enablers was integration, measured with the IPL-12 scale, which is an aggregate scale ranging from score 12–60, with higher scores indicative of a higher level of integration [29]. The IPL-12 scale is a short instrument that can be implemented across survey modes, and it applies to different groups of migrants, including new migrants, undocumented migrants and refugees. This scale is general enough to allow for meaningful comparisons across all migrant group and has no ethnic and language specifications [29]. Another enabler added for Indian migrants was the everyday discrimination scale (EDS) measured on a 5-point Likert, with eight items. The responses were dichotomized as ‘yes- discriminated (if response to any of the eight items was on 4 or 5 on Likert scale)’, ‘no- not being discriminated’ (if none of the responses to the items scored 4 or 5 on the Likert scale) [30].

Need factors included present oral health status as self-rated oral health in five categories which were dichotomized into good (excellent, good) or poor (fair, poor, very poor), bleeding gums (yes, no), and oral impact on daily performance (OIDP scale). This scale is a pre-validated questionnaire, measured on a six-point Likert scale reflecting the severity of the impact, ranging from 0 (indicating no impact), to 5 (indicating a very severe impact). Sum scores were created by adding the nine OIDP items as assessed originally. Finally, the OIDP frequency scores were dichotomized as either no (OIDP score of 0) or yes (with OIDP score of 1 or higher score) [31].

### Data analysis

We used IBM SPSS Statistics for Windows (Version 26.0) for the data analysis of this study. First, we performed simple descriptive analyses to describe the demographic characteristics of the two samples. These descriptive analyses were also done as a part of exploratory data analysis where we identified outliers, checked for normality of continuous variables and missing data. Eventually, missing data were handled through listwise deletion.

Then we performed univariable binary logistic regression analysis for the two outcome measures in both groups (Indian migrants and the host population) to get the unadjusted odds ratio. The significance level was set at 0.25. Before estimating the multiple logistic regression models, we examined collinearity amongst and between each predictor variable using the variance inflation factor (VIF). Only those variables with a VIF equal to or less than three were entered into the model. Multivariable binary logistic regression analysis with backward selection was then done to assess the independent associations (alpha < 0.05) between the two outcomes and the predictors with a p-value lower than 0.25 from the univariable analyses.

## Results

The final sample included 391 participants (147 Indian migrants and 244 from the host population). Table 1 outlines the descriptive composition of both groups. Indian migrants had a mean age of 38 years (median = 36 years, inter quartile range (IQR) = 32), consisted of 65% males, and the majority were married individuals (65%). The host population had a mean age of 42 years (median age = 43 years, IQR = 28), with a majority being married (80%) and females (60%). The frequency distribution of predisposing, enabling, and need factors of the Indian migrants and host population is described in Table 1. For the outcomes, only 51% of the Indian migrants visited an OHP for routine check-ups and preventive care, whereas 61% of Indian migrants were satisfied with OHPs. Among the host population, 91% visited an OHP for routine check-ups and preventive care, and 90% expressed satisfaction with OHPs.

**Table 1:** Descriptive characteristics of all the variables in both groups: Indian migrants and the host population

	Indian migrants (*n* = 147)	Host population (*n* = 244)
	n (%)	n (%)
***Predisposing factors***		
Age* n (mean, SD)	38.33 (8.98)	42.32 (14.60)
Gender		
*Males*	95 (65%)	98 (40%)
*Females*	52 (35%)	146 (60%)
Marital status		
*Married*	117 (80%)	159 (65%)
*Unmarried*	30 (20%)	85 (35%)
Internal LoC (mean, SD)	3.23 (0.31)	3.32 (0.35)
External LoC-Dentist (mean, SD)	2.38 (0.44)	2.24 (0.44)
External LoC-Chance (mean, SD)	1.62 (0.62)	1.67 (0.54)
External LoC- Social agents (mean, SD)	2.57 (0.71)	2.16 (0.75)
***Enabling resources***		
Income		
*Low income*	19 (13%)	62 (26%)
*Medium income*	29 (19%)	83 (32%)
*High income*	99 (68%)	99 (42%)
Education level		
*Low to medium*	6 (4%)	44 (18%)
*High*	141 (96%)	200 (82%)
Occupation*		
*Low paid jobs/unpaid*	19 (13%)	57 (24%)
*High paid jobs*	128 (87%)	181 (76%)
Dental insurance*		
*No*	71 (48%)	115 (48%)
*Yes*	76 (52%)	126 (52%)
Social support* (mean, SD)	35.33 (8.83)	36.05 (10.72)
Integration level (mean, SD)	40.31 (6.46)	-
Discrimination*		
*Yes*	33 (23%)	-
*No*	113 (77%)	-
***Need factors***		
Self-rated oral health*		
*Good*	87 (59%)	173 (71%)
*Fair to poor*	60 (41%)	70 (29%)
Gingival bleeding*		
*Yes*	39 (27%)	107 (44%)
*No*	104 (73%)	137 (56%)
OIDP impact		
*Yes impact*	109 (74%)	81 (33%)
*No impact*	38 (26%)	162 (67%)
***Outcome variables***		
Reason for dental visit*		
*Routine and preventive care*	75 (51%)	221 (91%)
*No visit/ pain/treatment*	72 (49%)	23 (9%)
†Satisfaction with oral health practitioners		
*Satisfied*	79 (61%)	213 (90%)
*Unsatisfied*	50 (39%)	23 (10%)

SD = Standard deviation, LoC = Locus of control, OIDP = Oral impact on daily performance

*Missing values: Observed between the range of 1 to 5 missing values in these variables

†Indian migrants (*n* = 18), who had never been to a dentist were excluded from the analysis of this variable

Table 2 displays the univariable analysis, computing crude odds ratios in both groups for the outcome ‘reasons for visiting an OHP’ (routine checkups and preventive care, or no visits or visit only for pain and treatment). Among the Indian migrants and host population, ten and five predictors had a p-value lower than 0.25, respectively. These predictors were incorporated into the subsequent multivariable analysis (Table 3). After entering the abovementioned ten predictors from Table 2 for the Indian migrants, three were significantly associated with visiting an OHP. Indians with higher scores on internal LoC [OR = 7.73 (95% CI:2.13;27.99)], external LoC (Dentist) [OR = 4.12 (95% CI:1.68;10.14)], and integration level [OR = 1.09 (95% CI:1.03;1.17)] had significantly higher odds of visiting an OHP for routine checkups and preventive care. For the host population, one out of the five predictors were seen to be significantly associated with this outcome. Having dental insurance was significantly associated with increased odds of visiting an OHP for routine checkups and preventive care [OR = 2.64 (95% CI:1.00;6.95] compared to no dental insurance.

**Table 2:** Univariable binary logistic regression (crude OR) of the reason for visiting an oral health professional (OHP) with predisposing, enabling, and need factors among the Indian migrants and the host population respectively

Outcome = Reason for visiting an oral health professional (OHP) No visits or visit only for pain and treatment = 0 (ref) Routine checkups and preventive care = 1				
	Indian migrants (*n*=147)		Host population (*n*=244)	
	Crude OR [95% CI]	p-value	Crude OR [95% CI]	p-value
***Predisposing factors***				
Age*	1.06 [1.02;1.11]	0.003	1.01 [0.98;1.04]	0.62
Gender				
*Males*	ref		ref	
*Females*	1.52 [0.77;2.99]	0.23	2.54 [1.05;6.12]	0.04
Marital status				
*Married*	ref		ref	
*Unmarried*	0.95 [0.43;2.12]	0.90	1.00 [0.41;2.47]	0.99
Internal LoC*	7.23 [2.31;22.65]	<0.001	0.77 [0.22;2.65]	0.68
External LoC-Dentist	3.53 [1.57;7.90]	0.002	1.32 [0.50;3.48]	0.57
External LoC- Chance	0.61 [0.36;1.04]	0.07	0.56 [0.25;1.25]	0.16
External LoC-Social agents*	0.93 [0.59;1.46]	0.75	0.86 [0.49;1.51]	0.59
***Enabling resources***				
Income				
*Low income*	ref		ref	
*Medium income*	2.25 [0.69;7.32]	0.18	1.22 [0.44;3.36]	0.70
*High income*	1.35 [0.50;3.64]	0.56	2.29 [0.76;6.97]	0.14
Education level				
*Low to medium*	ref		ref	
*High*	0.51 [0.09;2.86]	0.44	1.70 [0.63;4.59]	0.29
Occupation*				
*Low paid jobs/ Unpaid*	ref		ref	
*High paid jobs*	0.73 [0.27;1.93]	0.52	2.42 [0.98;6.01]	0.06
Dental insurance*				
*No*	ref		ref	
*Yes*	1.03 [0.54;1.96]	0.94	2.36 [0.92;6.06]	0.08
Social support	1.05 [1.01;1.09]	0.01	1.02 [0.98;1.06]	0.31
Integration [IPL-12] score	1.12 [1.06;1.19]	<0.001	NA	
Discrimination*				
*No*	ref		NA	
*Yes*	1.04 [0.48;2.27]	0.91		
***Need factors***				
Self-rated oral health*				
*Good*	ref		ref	
*Fair to poor*	0.42 [0.21;0.82]	0.01	0.92 [0.36;2.34]	0.86
Gingival bleeding*				
*No*	ref		ref	
*Yes*	0.74 [0.35;1.54]	0.41	1.24 [0.52;2.98]	0.63
OIDP impact*				
*No impact*	ref		ref	
*Yes impact*	0.51 [0.24;1.10]	0.09	0.69 [0.28;1.80]	0.43

LoC = Locus of control, OIDP = Oral impact on daily performance, NA = Not applicable

*Missing values: Observed between range of 1 to 6 missing values in these variables

**Table 3:** Multivariable binary logistic regression with backward selection for the outcome: the reason for visiting an oral health professional (OHP) among the Indian migrants and the host population respectively

Outcome = Reason for visiting an oral health professional (OHP) No visits or visit only for pain and treatment = 0 (ref) Routine checkups and preventive care = 1									
Variables included (9)	Model 1 Multivariable binary regression (backward selection) for the Indian migrants				Variables included (5)	Model 1 Multivariable binary regression (backward selection) for the Host population			
	Adjusted model with significant predictors from Table 2		Final adjusted model			Adjusted model with significant predictors from Table 2		Final adjusted model	
	95% CI	p-value	95% CI	p-value		95% CI	p-value	95% CI	p-value
***Predisposing factors***					***Predisposing factors***				
Age	1.04 [0.99;1.10]	0.12	NS		Gender				
Gender					*Males*	ref		NS	-
*Males*	ref		NS		*Females*	2.33 [0.91;5.98]	0.08		
*Females*	1.45 [0.61;3.47]	0.40			External LoC- Chance	0.59 [0.25;1.39]	0.23	NS	-
Internal LoC	5.87 [1.30;26.45]	0.02	7.73 [2.13;27.99]	0.002					
External LoC-Dentist	3.41 [1.33;8.77]	0.01	4.12 [1.68;10.14]	0.002					
External LoC-Chance	0.85 [0.43;1.69]	0.64	NS	-					
***Enabling resources***					***Enabling resources***				
Income					Income				
*Low*	ref		NS	-	*Low*	ref		NS	-
*Medium*	3.25 [0.69;15.31]	0.14			*Medium*	0.79 [0.25;2.49]	0.69		
*High*	1.12 [0.29;4.34]	0.87			*High*	1.32 [0.39;4.50]	0.66		
Integration [IPL-12] score	1.09 [1.01;1.17]	0.02	1.09 [1.03;1.17]	0.004	Occupation				
Social support	1.02 [0.97;1.07]	0.40	NS		*Low paid jobs/ unpaid*	ref		ref	
					*High paid jobs*	2.33 [0.85;6.41]	0.10	2.47 [0.94;6.49]	0.07
					Dental insurance				
					*No*	ref		ref	
					*Yes*	2.56 [0.97;6.78]	0.06	2.64 [1.00;6.95]	0.05
***Need factors***					***Need factors***				
Self-rated oral health									
*Good*	ref		NS	-					
*Fair to poor*	0.55 [0.23;1.29]	0.17							
OIDP impact									
*No impact*	ref		NS	-					
*Yes impact*	0.92 [0.34;2.45]	0.86							

NS = Not statistically significant, LoC = Locus of control, OIDP = Oral impact on daily performance

Table 4 presents the univariable analysis for being satisfied or not with OHPs in The Netherlands, revealing eleven and six predictors with a p-value below 0.25 among Indian migrants and the host population, respectively. These predictors were incorporated into the subsequent multivariable analysis (Table 5). Among Indians, the adjusted model shows that Indians who had low paid jobs [OR = 16.26 (95% CI:2.83;93.36)] and those with higher integration level [OR = 1.29 (95% CI:1.16;1.42)] had higher odds of being satisfied with OHPs. For the host population, the adjusted model shows that people with higher external LoC (Dentist) had significantly higher odds of being satisfied with the OHPs [OR = 2.85 (95% CI:1.19;6.88)] compared to those with lower scores.

**Table 4:** Univariable binary logistic regression (crude OR) of satisfaction with the oral health professionals (OHPs) in the Netherlands with predisposing, enabling, and need factors among the Indian migrants and the host population respectively

Outcome variable = Satisfaction with the oral health professionals (OHPs) in the Netherlands Unsatisfied = 0 (ref) Satisfied = 1				
	Indian migrants† (*n*=129)		Host population (*n*=244)	
	Crude OR [95% CI]	p-value	Crude OR [95% CI]	p-value
***Predisposing factors***				
Age*	1.06 [1.01;1.11]	0.01	1.04 [1.01;1.07]	0.01
Gender				
*Males*	ref		ref	
*Females*	1.57 [0.73;3.39]	0.25	1.27 [0.59;2.70]	0.54
Marital status				
*Married*	ref		ref	
*Unmarried*	0.68 [0.29;1.63]	0.40	0.39 [0.18;0.83]	0.02
Internal LoC*	2.41 [0.76;7.65]	0.14	0.97 [0.32;2.90]	0.95
External LoC- Dentist	2.17 [0.93;5.04]	0.07	2.55 [1.08;6.04]	0.03
External LoC- Chance	0.70 [0.39;1.24]	0.22	0.67 [0.33;1.35]	0.26
External LoC- Social agents	0.83 [0.50;1.37]	0.46	0.73[0.44;1.19]	0.20
***Enabling resources***				
Income				
*Low income*	ref		ref	
*Medium income*	0.49 [0.13;1.79]	0.28	1.11 [0.43;2.87]	0.83
*High income*	0.77 [0.24;2.41]	0.65	1.36 [0.53;3.49]	0.53
Education level				
*Low to medium*	ref		ref	
*High*	0.30 [0.03;2.66]	0.28	0.45 [0.13;1.55]	0.21
Occupation*				
*High paid jobs*	ref		ref	
*Low paid jobs/ Unpaid*	2.27 [0.69;7.39]	0.18	0.66 [0.28;1.55]	0.34
Dental insurance				
*Yes*	ref		ref	
*No*	1.31 [0.64;2.66]	0.46	0.83 [0.38;1.81]	0.65
Social support	1.04 [0.10;1.09]	0.06	1.00 [0.96;1.04]	0.92
Integration level	1.21 [1.11;1.31]	<0.001	NA	-
Discrimination*				
*No*	ref		NA	-
*Yes*	0.99 [0.42;2.33]	0.98		
***Need factors***				
Self-rated oral health*				
*Good*	ref		ref	
*Fair to poor*	0.43 [0.21;0.89]	0.02	1.19 [0.50;2.80]	0.69
Gingival bleeding*				
*No*	ref		ref	
*Yes*	0.60 [0.28;1.31]	0.20	0.60 [0.28;1.29]	0.19
OIDP impact *				
*No impact*	ref		ref	
*Yes impact*	0.31 [0.12;0.79]	0.01	0.85 [0.38;1.87]	0.68

*Missing values: Observed between range of 1 to 6 missing values in these variables

†Indian migrants (*n* = 18), who had never been to a dentist were excluded from the analysis of this variable

NA = Not applicable for the host population, LoC = Locus of control, OIDP = Oral impact on daily performance

**Table 5:** Multivariable binary logistic regression with backward selection for the outcome: satisfaction with oral health professionals (OHPs) in the Netherlands among the Indian migrants and the host population respectively

Outcome variable = Satisfaction with the oral health professionals (OHP) in the Netherlands Unsatisfied = 0 (ref) Satisfied = 1									
Variables recorded. (11)	Model 2 Multivariable binary regression (backward selection) for the Indian migrants				Variables recorded. (6)	Model 2 Multivariable binary regression (backward selection) for the Host population			
	Adjusted model with significant predictors from Table 4		Final adjusted model			Adjusted model with significant predictors from Table 4		Final adjusted model	
	95% CI	p-value	95% CI	p-value		95% CI	p-value	95% CI	p-value
***Predisposing factors***					***Predisposing factors***				
Age	1.02 [0.96;1.09]	0.46	NS	-	Age	1.02 [0.99;1.05]	0.30	NS	-
Gender					Marital status				
*Males*	ref		NS	-	*Married*	ref		ref	
Females	0.89 [0.28;2.89]	0.86			*Unmarried/ single*	0.43 [0.19;1.00]	0.05	0.34 [0.16;0.75]	0.007
Internal LoC	1.54 [0.28;8.32]	0.62	NS	-	External LoC-Dentist	3.07 [1.19;7.93]	0.02	2.86 [1.19;6.88]	0.02
External LoC- Dentist	1.66 [0.56;4.90]	0.36	NS	-	External LoC- Social agents	0.73 [0.43;1.23]	0.24	NS	-
External LoC- Chance	0.92 [0.41;2.07]	0.83	NS	-					
***Enabling resources***					***Enabling resources***				
Occupation					Education				
*High paid jobs*	ref		ref		*Low to medium*	ref		NS	-
*Low paid jobs/ unpaid*	16.15 [2.16;120.74]	0.007	16.26 [2.83;93.36]	0.002	*High*	0.54 [0.15;1.95]	0.35		
Social support	1.02 [0.96;1.08]	0.60	NS						
Integration [IPL-12]	1.26 [1.13;1.40]	<0.001	1.29 [1.16;1.42]	< 0.001					
***Need factors***					***Need factors***				
Self-rated oral health					Bleeding gums				
*Good*	ref				*No*	ref			
*Fair to poor*	0.71 [0.26;1.97]	0.51	NS	-	*Yes*	0.62 [0.28;1.39]	0.25	NS	-
Bleeding gums									
*No*	ref		NS	-					
*Yes*	1.11 [0.38;3.22]	0.85							
OIDP Impact									
*No impact*	ref		NS	-					
*Yes impact*	0.60 [0.18;2.00]	0.41							

*Indian migrants (*n* = 18), who had never been to a dentist were excluded from the analysis of this variable

NS = Not statistically significant, LoC = Locus of control, OIDP = Oral impact on daily performance

**Fig. 2:**
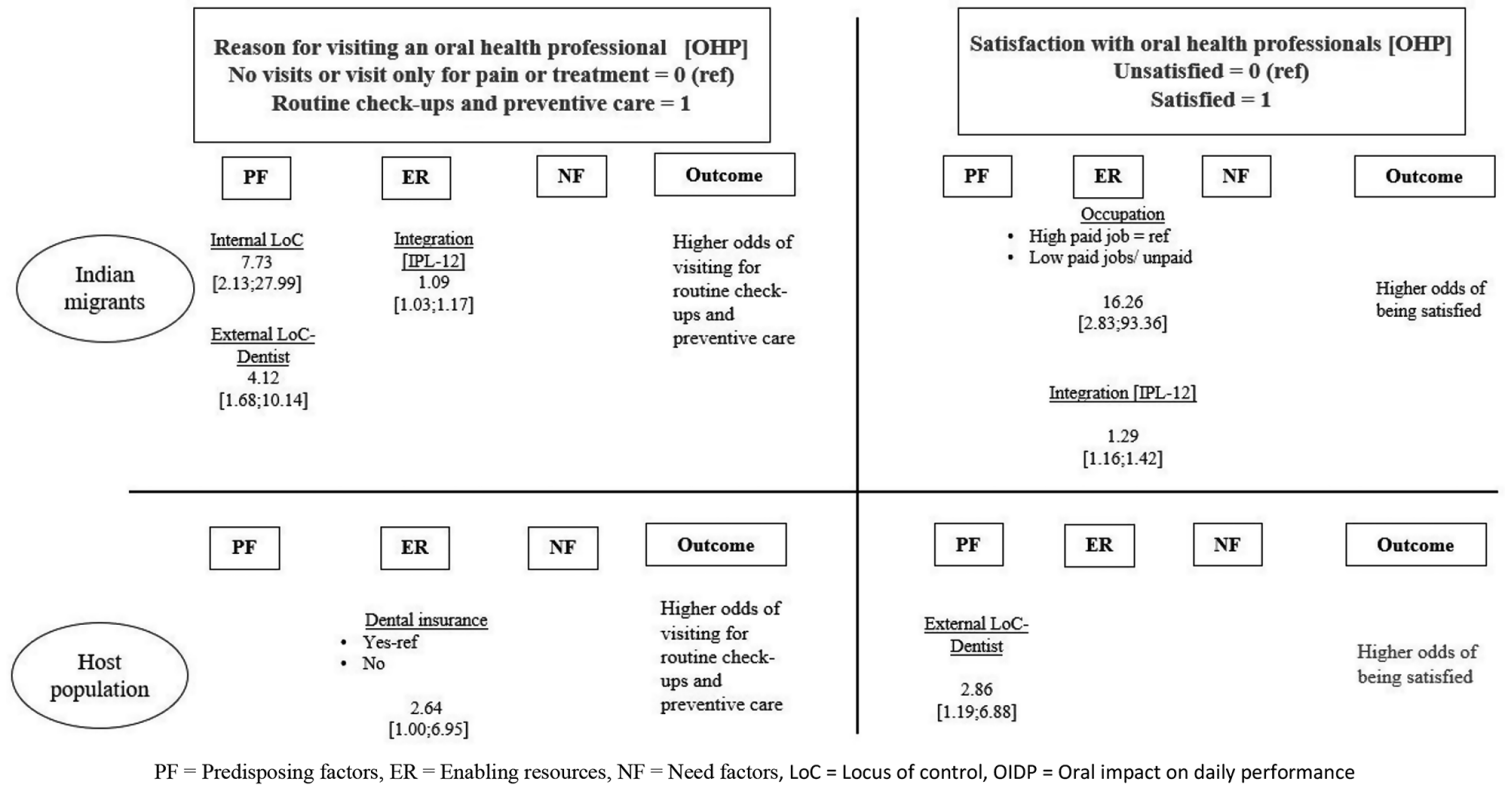
An overall summary of the significant predictors of oral healthcare utilization and satisfaction among Indian migrants and the host population in the Netherlands based on Andersen’s behavioural model for healthcare utilization (adapted). PF = Predisposing factors, ER = Enabling resources, NF = Need factors, LoC = Locus of control, OIDP = Oral impact on daily performance

Figure 2 shows the diagrammatic representation of the results as seen in the models using multivariable regression analyses for both the outcomes in the two groups. Different predictor variables were associated with the reasons for visiting an OHP among both the groups. For satisfaction with the OHPs, only predisposing factors were significantly associated with the outcome among the host population, while both predisposing and enabling resources were associated among the Indian migrants.

## Discussion

This study explored the predictor variables, including predisposing, enabling and need factors, associated with reasons for visiting an OHP and satisfaction with OHPs in the Netherlands, among the Indian migrants and the host population in the Netherlands. Indians predominantly visited an OHP for routine check-ups and preventive care due to predisposing factors such as internal belief and the trust in dentist. In addition, integration was seen as a significant enabler as well. Whereas in the host population a different enabling resource, having dental insurance, was significantly associated with reasons for routine check-ups and preventive visits. Furthermore, satisfaction with OHPs among Indians was associated with enabling resources such as integration and low-paid jobs. Conversely, the host population’s satisfaction was primarily associated with predisposing factors, notably higher LoC in the dentist.

Our multivariable analysis revealed that Indians with higher internal LoC and higher trust in the dentist demonstrated significantly higher odds of visiting for routine checkups and preventive care. While prior studies have linked internal LOC beliefs to regular dental attendance [26], our study extends this understanding, suggesting that Indians with high internal belief and those who value the expertise of the dentist are more likely to visit the dentist for routine check-ups. However, the existing literature examining the correlation between LoC and the utilization of dental services is limited, particularly in the context of the Netherlands. Most studies concentrate on the LoC among migrant parents and its implications for dental caries among children [27, 32]. Consequently, our ability to delve deeper into the significance of LoC within the host population remains constrained, highlighting a notable gap in understanding the broader dynamics of this psychological factor in shaping oral healthcare behaviours.

Furthermore, our findings revealed that higher integration levels among Indian migrants were a significant enabler for their proactive engagement with dental care. This aligns with broader research trends observed in studies on migrants, indicating that acculturation, measured through indicators like language proficiency and length of stay, has a positive role in dental care utilization [33]. Specifically. among Indian migrants, systematic reviews have observed that highly acculturated Asian migrants, including Indians, are more likely to utilize dental services in the host countries [11]. While our findings shed light on these associations, further research is needed to delve deeper into the nuanced interplay between psychological factors, acculturation, and oral healthcare care utilization among migrant populations.

Among the host population, those having dental insurance had higher odds of visiting an OHP for routine checkups and preventive care. This aligns with the findings reported by CBS, indicating that 59% of the Dutch population had dental insurance in 2020/2021, and those with insurance were more inclined to seek oral healthcare compared to those without coverage [34, 35]. It has been well documented that socioeconomic position is related to not only with poor oral health outcomes, but also with lower use of dental services [36, 37]. Hence it is not surprising to see the direction of this finding especially since dental insurance is coupled with purchasing power and higher socioeconomic position would enable dental visits [38, 39]. Interestingly, dental insurance did not emerge as a significant predictor among the Indian migrants. This may be due to other factors such as cultural perceptions, language barriers, or varying oral healthcare-seeking behaviours play more important roles, necessitating a nuanced understanding to unravel the intricacies of their distinct oral healthcare patterns.

Satisfaction with healthcare, including oral health, is vital, reflecting how well healthcare providers meet patient needs and earn their trust [40]. In our study, we found that Indians, with low-income jobs and higher levels of integration had higher odds of being satisfied with OHPs. Studies in India have suggested that Indians with some forms of financial stability are usually content with oral care providers [41–43]. The most frequent explanation cited a better understanding of the treatment plan, and trust in the advice of the dentist. As Indians move to high-income countries, integration, a component of acculturation, likely influences satisfaction due to differences in oral healthcare infrastructure and access. Even among the host population, higher trust in the dentist was a significant factor influencing satisfaction with OHPs. Although direct studies on LoC scales and satisfaction with OHPs are lacking, Peker et al. [26]. observed similar trends, emphasizing the value of considering such factors in planning oral health interventions. However, the sparse data necessitates further research to comprehensively understand the role of social and environmental determinants, including integration, in influencing satisfaction levels with OHPs in migrant populations.

Lastly, in our study, the need factors did not show significant associations with any outcome, contrary to findings in many studies utilizing the Andersen Model, where perceived needs often influence dental visit frequency. The absence of this association in our research highlights the complex interplay of factors affecting dental healthcare decisions among our participants. Potential explanations might include cultural differences in how individuals perceive their oral health needs, variations in access to information, or unique healthcare-seeking behaviours within our study population. Further qualitative research could explore these nuances, providing a deeper understanding of the intricacies behind these unexpected findings and shedding light on the factors that shape oral health decision-making among our participants.

Our study is not without limitations. A notable challenge was the low response rate (13.5%), which may have introduced selection bias, thereby limiting the generalizability of our findings. Additionally, this resulted in wider confidence intervals and skewed distribution of some variables. Also, the predominant representation of participants from higher education and higher income strata in both groups could introduce a bias toward individuals with elevated educational and economic backgrounds. Moreover, this study was conducted during the COVID-19 lockdown. Hence, additional strategies to improve representation among migrant population were restricted [44]. In addition, COVID may have also impacted the self-reported oral health needs of people in general, which could similarly have influenced our study population as well [45, 46]. Despite this limitation, our study does contribute to bridge the gap in literature, by shedding light on the present status of oral health utilization patterns among Indian migrants in the Netherlands. Self-reported oral health data, gathered through questionnaires, could be influenced by subjective and recall biases, impacting result accuracy [47]. The study’s cross-sectional design prevents establishing temporal relationships. Furthermore, the homogeneity in our sample, while enhancing specific insights into Indian migrants, limits the broader applicability of the findings to more diverse migrant populations. Also, we included only Indian migrants who have lived in the Netherlands for five years or more, ensuring that the participants had a stable residence in the country. As a result, we cannot make inferences to the individuals who live part-time between India and the Netherlands.

A key strength lies in our specific focus on Indian migrants from the Indian subcontinent, ensuring cultural homogeneity and providing unique insights into this particular group’s oral healthcare utilization patterns. Unlike convenience sampling often used in similar studies, our research employed a stratified random sampling method, possibly reducing selection bias. Furthermore, incorporating variables like LoC and integration, deepened the analysis of psychosocial factors affecting oral healthcare use, enhancing the study’s depth and richness. Despite limited previous studies in this area, our research contributes valuable insights into the oral health dynamics of Indian migrants in the Netherlands.

In conclusion, our findings emphasize the need for incorporating different research designs, such as qualitative exploration to better understand the unique oral healthcare-seeking behaviours within the migrant groups in the host country. Additionally, longitudinal studies, tracking the influence of predisposing, enabling and need factors over time and their impact on oral health decisions would enrich our understanding of these complex dynamics. This would enable more targeted interventions addressing awareness about insurance benefits to all. Since integrated Indians are more inclined to utilize dental care services, policymakers can leverage this resource to enhance accessibility to dental care for migrants who integrate effectively in the host society. This may not only lead to improved individual oral health outcomes but also reduce the burden on the oral healthcare system. Additionally, healthier migrant populations contribute positively to the host society’s overall public health, fostering social cohesion and integration. Moving forward, OHPs in the Netherlands can tailor their communication approaches based on the abovementioned points so that they can adapt to more culturally sensitive interactions, encouraging trust and positive patient experiences.

## Electronic supplementary material

Below is the link to the electronic supplementary material.

Supplementary Material 1

Supplementary Material 2

## Data Availability

The data underlying the results presented in the study are available from: https://figshare.com/s/c6a11ff0d50ae082e770.

## Abbreviations

OHP: Oral healthcare professional
