# Supplementary material for: Predictors of oral healthcare utilization and satisfaction among Indian migrants and the host population in the Netherlands
Source: BMC Oral Health. 2024 Oct 15;24:1228. doi: 10.1186/s12903-024-04988-y (PMC11481359; doi:10.1186/s12903-024-04988-y)
Supplement: Supplementary file 1 — Supplementary Material 1 [file 12903_2024_4988_MOESM1_ESM.pdf]

ID-CODE

MONDGEZONDHEID, MONDHYGIENE EN TANDARTSBEZOEK VAN INDIANSE MIGRANTEN IN NEDERLAND

VRAGENLIJST

TOESTEMMINGSFORMULIER

Mondgezondheid, mondhygiëne en tandartsbezoek van Indiase migranten die in Nederland wonen, in vergelijking met de Nederlandse bevolking

- Ik heb de informatiebrief over dit onderzoek gelezen. Ik heb de mogelijkheid gekregen om vragen te stellen. Mijn vragen zijn beantwoord. Ik heb genoeg tijd gekregen om te beslissen of is mee wil doen.
- Ik begrijp dat deelname volledig vrijwillig is.
- Ik begrijp dat ik het recht heb om op ieder moment af te zien van deelname zonder daarbij een reden te hoeven opgeven.
- Ik begrijp dat alle gegevens volledig vertrouwelijk zullen worden verwerkt.
- Ik geef toestemming om deel te nemen aan dit onderzoek. Ik geef daarmee toestemming om mijn gegevens uit de vragenlijst te gebruiken voor de doelstelling van het onderzoek.

☐ Ik geef toestemming voor deelname

☐ Ik geef geen toestemming voor deelname

Handtekening ___________________________

Datum _______________________________

VU VRIJE UNIVERSITEIT AMSTERDAM

UNIVERSITEIT VAN AMSTERDAM

ACADEMISCH CENTRUM TANDHEELKUNDE AMSTERDAM

ACTA

GUSTAV MAHLERLAAN 3004
1081 LA AMSTERDAM

VRAGENLIJST – MONDGEZONDHEID VAN INDIASE

MIGRANTEN IN

U dient deze vragenlijst voor uzelf in te vullen.

- Neem de tijd en lees de vragen aandachtig door. Als u de vragen niet begrijpt kunt u iemand vragen om u te helpen. Als u hulp vraagt is het belangrijk dat u wel uw eigen antwoord invult. U kunt voor hulp ook contact opnemen met de onderzoeker (telefoon: 0626870875).
- Als u niet zeker weet hoe u de vraag moet beantwoorden, probeer dan het antwoord te geven dat het beste bij u past. Het is belangrijk om alle vragen te beantwoorden. Alleen als u bij een antwoord wordt gevraagd om naar een specifieke vraag te gaan die zich verderop in de vragenlijst bevindt, dan kunt u de vragen daartussen overslaan.
- U hoeft de vragenlijst niet in één keer in te vullen. U kunt een deel invullen en het later afmaken.
- Het invullen van de vragenlijst duurt ongeveer 20-30 minuten. Alle vragen zijn belangrijk, want deze helpen ons om inzicht te krijgen in uw mondgezondheid en uw dagelijkse activiteiten gerelateerd aan uw (mond)gezondheid.
- Er zijn geen ‘goede’ of ‘foute’ antwoorden; wel is het belangrijk om alle vragen naar waarheid in te vullen.
- Deze vragenlijst is online beschikbaar via **https://tiny.cc/qna5tz.** Als u deze vragenlijst online wilt invullen, kopieer dan deze link op uw computer en u wordt doorgestuurd naar de online vragenlijst.
- U kunt ook de QR-code gebruiken en de code scannen. Dit brengt u ook naar de digitale vragenlijst.
- Als u deze papieren versie van de vragenlijst invult, dan kunt u deze opsturen met de bijgeleverde retourenvelop. Een postzegel is niet nodig.

1. ALGEMENE INFORMATIE

1.1 Wat is uw geslacht?

- ☐ Man
- ☐ Vrouw
- ☐ Anders

1.2 Wat is uw leeftijd? ________jaar

1.3 Wat is uw burgerlijke staat?

- ☐ Gehuwd / geregistreerd partnerschap
- ☐ Samenwonend
- ☐ ongehuwd / nooit gehuwd geweest
- ☐ Gescheiden
- ☐ Weduwe

> Categories were condensed to:
> 1=Married/ cohabiting
> 2=Single/divorced/widow

1.4 Hoeveel mensen wonen er in uw huishouden (inclusief uzelf)? Als u alleen woont vult u '1' in. ______________

1.5 Wat is uw nationaliteit (zoals vermeld in uw paspoort)?___________________________

1.6 Wat is uw postcode (alleen de eerste vier cijfers)? ______________________________

2. LEEFSTIJL EN GEWOONTEN

2.1 Rookt u?

- ☐ Ja
- ☐ Nee, ik heb nooit gerookt
- ☐ Nee, maar ik heb vroeger gerookt

> Categories were condensed to:
> 0= No/ I quit
> 1= Yes

2.2 Hoe vaakt gebruikt u rookvrije vorm van tabak?

- ☐ Nooit
- ☐ Minder dan 1 keer per maand
- ☐ 1 keer per maand
- ☐ 2-4 keer per maand
- ☐ 2-4 keer per week
- ☐ 5-6 keer per week
- ☐ Dagelijks

This question was not used in analysis, since Dutch do not use chewing form of tobacco.

2.3 Hoe vaak drinkt u alcoholhoudende drank?

- ☐ Ik drink geen alcohol
- ☐ Minder dan 1 keer per maand
- ☐ 1 keer per maand
- ☐ 2-4 keer per maand
- ☐ 2-4 keer per week
- ☐ 5-6 keer per week
- ☐ Dagelijks

Categories were condensed to:
0=Never
1= Monthly
2=Weekly

2.4 Hoe vaak eet u zoetigheden, zoals koekjes of chocola?

- ☐ Zelden tot nooit
- ☐ Minder dan 1 keer per maand
- ☐ 1 keer per maand
- ☐ 2-4 keer per maand
- ☐ 2-4 keer per week
- ☐ 5-6 keer per week
- ☐ Dagelijks

Categories were condensed to:
1=Monthly
2=Weekly
3=Daily

2.5 Hoe vaak drinkt u frisdrank?

- ☐ Zelden tot nooit
- ☐ Minder dan 1 keer per maand
- ☐ 1 keer per maand
- ☐ 2-4 keer per maand
- ☐ 2-4 keer per week
- ☐ 5-6 keer per week
- ☐ Dagelijks

Categories were condensed to:
0=Never
1=Monthly
2=Weekly

2.6 Voegt u suiker toe aan warme dranken?

- ☐ Ja, altijd
- ☐ Ja, soms
- ☐ Nee, nooit

Categories were condensed to:
0=Never
1=Yes

De volgende vragen gaan over uw gewoonten om uw mond en gebit schoon te maken.

2.7 Wat gebruikt u om uw gebit schoon te maken? (*U kunt meerdere antwoorden aankruisen)*

- ☐ Handtandenborstel
- ☐ Electrische tandenborstel
- ☐ Tandpasta
- ☐ Anders, namelijk ____

2.8 Hoe vaak gebruikt u de volgende reinigingsmiddelen?

		Dagelijks	Een peer keer per week	1 keer per week	Elke 2 weken	1 keer per maand	Nooit
a	Tandenstokers	☐	☐	☐	☐	☐	☐
b	Flosdraad	☐	☐	☐	☐	☐	☐
c	Reiningsborsteltjes (voor tussen de tanden)	☐	☐	☐	☐	☐	☐
d	Mondwater	☐	☐	☐	☐	☐	☐
e	Anders, namelijk ___________________	☐	☐	☐	☐	☐	☐

2.9 Gebruikt u tandpasta met fluoride?

☐ Ja

☐ Nee

☐ Weet ik niet

Categories were condensed to:
0=Yes
1=No/I do not know

2.10 Hoeveel vertrouwen heeft u erin dat u uw tanden poetst in de volgende omstandigheden?

This question was not used for analysis

		Veel vertrouwen	Redelijk vertrouwen	Niet zo veel vertrouwen	Helemaal geen vertrouwen
a	Als u moe bent in de avond	☐	☐	☐	☐
b	Als u niet naar de tandarts hoeft in de komende tijd	☐	☐	☐	☐
c	Als u op vakantie bent	☐	☐	☐	☐
d	Als u veel werk te doen heeft	☐	☐	☐	☐
e	Als u hoofdpijn heeft of ziek bent	☐	☐	☐	☐

2.11 Hoe beoordeelt u de volgende stellingen?

This question was not used for analysis

		Helemaal niet mee eens	Niet mee eens	Eens	Helemaal mee eens
a	Wat ik ook doe, ik krijg toch wel kiespijn, want ik kan mijn tanden niet veranderen.	☐	☐	☐	☐
b	Ik kan gaatjes en tandvleesproblemen voorkomen als ik regelmatig poets.	☐	☐	☐	☐
c	Alleen met het advies van mijn tandarts kan ik mijn mondhygiëne op peil houden.	☐	☐	☐	☐
d	Ik denk dat de achteruitgang van mijn mondgezondheid een kwestie is van pech.	☐	☐	☐	☐
e	Ik ga onmiddellijk naar de tandarts wanneer ik merk dat er iets mis is met mijn mondgezondheid.	☐	☐	☐	☐
f	De waarschuwingen van mijn familie hebben grote invloed op mijn mondgezondheid.	☐	☐	☐	☐
g	Als ik geluk heb zal mijn mondgezondheid snel verbeteren.	☐	☐	☐	☐
h	Ik weet wat ik moet doen om mijn mond en gebit gezond te houden.	☐	☐	☐	☐
i	De waarschuwingen van mijn vrienden of mensen in mijn directe omgeving hebben een grote invloed op mijn mondgezondheid.	☐	☐	☐	☐
j	Het is toeval als mijn mondgezondheid achteruit gaat.	☐	☐	☐	☐
k	Ik ben direct verantwoordelijk voor mijn eigen mondgezondheid.	☐	☐	☐	☐
l	Mijn mondgezondheid is afhankelijk van de tandarts.	☐	☐	☐	☐
m	Als mijn mondgezondheid verslechtert, laat ik alles op zijn beloop en zeg ik: "Ik zal dragen wat ik moet lijden".	☐	☐	☐	☐
n	Ik geef mezelf de schuld als ik gaatjes krijg.	☐	☐	☐	☐

		**Helemaal niet mee eens**	**Niet mee eens**	**Eens**	**Helemaal mee eens**
o	De tandarts onderhoud mijn mondgezondheid.	☐	☐	☐	☐
p	Wat ik ook doe, mijn tandvlees zal ontstoken zijn en blijven bloeden, want ik kan mijn tandvlees niet veranderen.	☐	☐	☐	☐
**q**	De enige manier om mijn mondgezondheid te behouden is door zelf voor mijn mond en gebit te zorgen.	☐	☐	☐	☐
**r**	Ik heb de afwezigheid van gaatjes en tandvleesproblemen en de verbetering van mijn mondgezondheid te danken aan mijn tandarts.	☐	☐	☐	☐
**s**	Mijn tanden zullen voor een langere periode gezond blijven als ik ze goed verzorg.	☐	☐	☐	☐
**t**	Ik voorkom gaatjes en tandvleesziekten als ik goed voor mijn mondgezondheid zorg.	☐	☐	☐	☐
**u**	Ik doe alleen wat de tandarts mij vertelt.	☐	☐	☐	☐
**v**	Ik weet dat ik de zorg voor mijn gebit heb verwaarloosd als ik problemen krijg met mijn mondgezondheid.	☐	☐	☐	☐
**w**	Geluk speelt een grote rol in de snelheid waarmee mijn gaatjes en tandvleesproblemen zullen herstellen.	☐	☐	☐	☐
**x**	Ik zorg goed voor het onderhoud en de verbetering van mijn mondgezondheid.	☐	☐	☐	☐
**y**	Het is mogelijk om op de lange termijn gezonde tanden te hebben als ik doe wat er moet gebeuren.	☐	☐	☐	☐
**z**	De enige manier om mijn mondgezondheid te behouden is door de aanbevelingen van de tandarts op te volgen.	☐	☐	☐	☐

3: MONDGEZONDHEID

3.1 Hoe beoordeelt u de gezondheid van uw mond (tanden, kiezen, tandvlees)?

- ☐ Uitstekend
- ☐ Goed
- ☐ Redelijk
- ☐ Slecht
- ☐ Zeer slecht

Categories were condensed to:
0=Very good to good
1= Fair to poor

3.2 Volwassenen kunnen 32 tanden en kiezen hebben (inclusief verstandskiezen), maar deze kunnen over de tijd verloren gaan.

Tel het aantal eigen tanden in uw bovenkaak (graag een getal tussen de 0 en 16 invullen) ________________________

Tel het aantal eigen tanden in uw onderkaak (graag een getal tussen de 0 en 16 invullen) ________________________

3.3 Heeft u een kunstgebit in uw bovenkaak?

- ☐ Ja een volledig kunstgebit
- ☐ Ja een gedeeltelijk kunstgebit
- ☐ Nee

Categories were condensed to:
0=No
1=Yes

3.4 Heeft u een kunstgebit in uw onderkaak?

- ☐ Ja een volledig kunstgebit
- ☐ Ja een gedeeltelijk kunstgebit
- ☐ Nee

Categories were condensed to:
0=No
1=Yes

3.5 Heeft uw tandarts of mondhygiënist u ooit verteld dat u tandvleesproblemen (parodontale problemen) heeft?

- ☐ Ja
- ☐ Nee
- ☐ Weet ik niet

Categories were condensed to:
0=No/ I do not know
1=Yes

3.6 Heeft u last van loszittende tanden?

- ☐ Nee
- ☐ Ja, enigszins loszittende tanden
- ☐ Ja, erg loszittende tanden

Categories were condensed to:
0=No
1=Yes

3.7 Wat zou u doen als u kiespijn heeft door aanhoudende zwelling?

- ☐ Proberen de pijn tegen te gaan met pijnstillers of medicijnen en wachten tot de pijn over gaat
- ☐ Direct laten behandelen
- ☐ Bespreken met de familie en beslissen op basis van hun advies
- ☐ Wachten met behandeling totdat u uw geboorteland bezoekt, bijvoorbeeld voor vakantie
- ☐ Anders, namelijk ____________________

3.8 Hoe vaak heeft u in de afgelopen 3 maanden last gehad van de onderstaande klachten

		**Nooit**	**Minder dan 1 keer per maand**	**1-3 keer per maand**	**1-2 keer per week**	**3-4 keer per week**	**Bijna dagelijks / dagelijks**
**a**	Kiespijn	☐	☐	☐	☐	☐	☐
**b**	Bloedend of gezwollen tandvlees	☐	☐	☐	☐	☐	☐

3.8 Categories were condensed to:
0=Never
1=Yes

3.9 De volgende vraag gaat over problemen die u kunt ervaren door uw klachten van uw mond, uw gebit en/of uw prothese. Hoeveel invloed hebben de onderstaande problemen gehad op uw dagelijkse leven in de afgelopen 3 maanden op een schaal van 0 tot 5 (0 is helemaal geen invloed en 5 is extreem veel invloed).

		**Geen invloed**					**Extreem veel invloed**
a	Problemen met eten	0 ☐	1 ☐	2 ☐	3 ☐	4 ☐	5 ☐
b	Problemen met spreken / praten	0 ☐	1 ☐	2 ☐	3 ☐	4 ☐	5 ☐
c	Problemen met het reinigen van uw gebit / prothese	0 ☐	1 ☐	2 ☐	3 ☐	4 ☐	5 ☐
d	Problemen met uitgaan, bijvoorbeeld naar de winkel gaan of op bezoek gaan bij iemand	0 ☐	1 ☐	2 ☐	3 ☐	4 ☐	5 ☐
e	Problemen met ontspannen (inclusief slapen)	0 ☐	1 ☐	2 ☐	3 ☐	4 ☐	5 ☐
f	Problemen met (glim)lachen en het laten zien van uw gebit zonder schaamte	0 ☐	1 ☐	2 ☐	3 ☐	4 ☐	5 ☐
g	Problemen met uitvoeren van uw werk of functie	0 ☐	1 ☐	2 ☐	3 ☐	4 ☐	5 ☐
h	Problemen met uw emotionele stabiliteit, bijvoorbeeld eerder boos worden dan normaal	0 ☐	1 ☐	2 ☐	3 ☐	4 ☐	5 ☐
l	Problemen met het plezier hebben van sociaal contact, zoals met familieleden, vrienden of buren	0 ☐	1 ☐	2 ☐	3 ☐	4 ☐	5 ☐

This is the question on OIDP (Oral impact on daily performance) scale. We used the dichotomized variable as: Has OIDP impacted your life?
0=No
1=Yes

4: TANDARTSBEZOEK

4.1 Heeft u voor de COVID- lockdown, in 2019, een tandarts bezocht?

- ☐ Ja
- ☐ Nee
- ☐ Kan ik mij niet herinneren

Categories were condensed to: Have you visited any dental professional before COVID 19 lockdown?
0=No
1=Dentist only
3=Both
2=Hygienist only- This was taken out as only 5 Indians said they had visited a hygienist only

4.2 Heeft u voor de COVID- lockdown een mondhygiënist bezocht?

- ☐ Ja
- ☐ Nee
- ☐ Kan ik mij niet herinneren

4.3 Wat is Meestal voor u de reden om naar de tandarts te gaan? (U kunt meerdere antwoorden aankruisen)

- ☐ Consult of advies
- ☐ Pijn of problemen met de tanden, tandvlees of mond
- ☐ Behandeling of vervolgbehandeling
- ☐ Een controle
- ☐ Kan ik mij niet herinneren
- ☐ Een andere reden, namelijk ____________________

4.4 Hoe ver is de tandarts bij uw huis vandaan?

- ☐ In dezelfde buurt
- ☐ Minder dan 30 minuten reizen
- ☐ Tussen 30 minuten en 1 uur reizen
- ☐ Meer dan een uur reizen
- ☐ Weet ik niet

This question was not used for analysis

4.5 Heeft u voor de COVID- lockdown de volgende behandelingen gehad?

		Ja	Nee	Kan ik mij niet herinneren
**a**	Behandeling voor tandvleesproblemen (parodontitis)	☐	☐	☐
**b**	Nieuwe vulling	☐	☐	☐
**c**	Wortelkanaalbehandeling	☐	☐	☐
**d**	Trekken van een tand of kies	☐	☐	☐
**e**	Nieuwe vervangende tand of kies (kroon, brug of implantaat)	☐	☐	☐
**f**	Reinigen van het gebit	☐	☐	☐
**g**	Behandeling puur om de esthetiek te verbeteren (behandeling van de voorste tanden, bleken, orthodontie)	☐	☐	☐

4.6 Hoe tevreden bent u met uw tandarts?

- ☐ Erg tevreden
- ☐ Redelijk tevreden
- ☐ Neutraal
- ☐ Redelijk ontevreden
- ☐ Erg ontevreden

Categories were condensed to:
1=Satisfied
2=Neutral
3=Unsatisfied

4.7 Als u nog nooit de tandarts hebt bezocht, wat is de reden hiervoor? (U kunt meerdere antwoorden aankruisen). Als u de vragen 5.1 t/m 5.7 heeft ingevuld, ga dan door naar vraag 5.9

- ☐ Er is niks mis met mijn tanden
- ☐ Tandartsangst of verwaarlozing
- ☐ Gebrek aan vertrouwen in de tandartspraktijk
- ☐ Het probleem verdwijnt in de loop van tijd
- ☐ Tandartsbezoeken kosten veel geld
- ☐ Anders, namelijk ______________________________

4.8 Heeft u een tandartsverzekering?

- ☐ Ja
- ☐ Nee
- ☐ Weet ik niet

Categories were condensed to:
1=Yes
2=No/ I do not know

5. ALGEMENE GEZONDHEID

5.1 Hoe beoordeelt u uw gezondheid?

This section was not used for analysis

- ☐ Uitstekend
- ☐ Goed
- ☐ Redelijk
- ☐ Slecht
- ☐ Zeer slecht

5.2 Heeft u last van de volgende gezondheidsproblemen?

		Ja	Nee
a	Diabetes	☐	☐
b	Hoge bloeddruk	☐	☐
c	Ernstige hartaandoening (bijvoorbeeld hartfalen of angina pectoris)	☐	☐
d	Kanker	☐	☐
e	Anders	☐	☐

Als u ‘anders’ heeft aangekruist, noem hier het gezondheidsprobleem waar het om gaat

____

De volgende vragen hoeft u alleen te beantwoorden als u gediagnosticeerd bent met diabetes. Anders kunt u doorgaan naar vraag 6.1**.**

5.3 Bent u in de afgelopen 3 jaar getest op hoge bloeddruk?

- ☐ Ja
- ☐ Nee

5.4 Op welke leeftijd bent u gediagnosticeerd met diabetes? Als u het niet precies weet, probeer dan een schatting te maken. _____________jaar oud.

5.5 Is er iemand in uw directe familie (ouders, broers, zussen of kinderen) gediagnosticeerd met diabetes?

- ☐ Ja
- ☐ Nee
- ☐ Weet ik niet

5.6 Bent u in de afgelopen 12 maanden door een dokter behandeld voor diabetes?

- ☐ Ja
- ☐ Nee

6: SOCIALE STEUN

6.1 Wat voor soort steun krijg je meestal?

		Altijd	Meestal	Vaak	Soms	Nooit	Niet van toepassing
a	Iemand die je liefde en affectie geeft	☐	☐	☐	☐	☐	☐
b	Iemand met wie je een leuke dag kan beleven	☐	☐	☐	☐	☐	☐
c	Iemand die je kan vertrouwen en mee kan praten over je problemen/gevoelens	☐	☐	☐	☐	☐	☐
d	Iemand waarmee je kan ontspannen	☐	☐	☐	☐	☐	☐
e	Iemand die voor je kan koken als je het even niet zelf kan	☐	☐	☐	☐	☐	☐
f	Iemand die je kan helpen met je dagelijkse werkzaamheden wanneer je ziek bent	☐	☐	☐	☐	☐	☐
g	Iemand met wie je je persoonlijke problemen en angsten kan delen	☐	☐	☐	☐	☐	☐
h	Iemand waar je naar toe kan gaan voor suggesties hoe om te gaan met persoonlijke problemen	☐	☐	☐	☐	☐	☐
i	Iemand van wie je houdt en je het gevoel geeft dat je gewaardeerd wordt	☐	☐	☐	☐	☐	☐

This section was not used for analysis

7: OPLEIDING EN BEROEP

7.1 In welk land bent u geboren?

- ☐ Nederland
- ☐ Anders, namelijk ____

7.2 Wat is de hoogste opleiding die u in Nederland heeft voltooid? Het gaat om de hoogste opleiding die u hebt afgerond met diploma of voldoende getuigschrift.

- ☐ Geen (geen onderwijs gevolgd, het onderwijs niet afgemaakt, of opleiding in ander land afgerond)
- ☐ Lager onderwijs (basisschool, speciaal basisonderwijs)
- ☐ Lager of voorbereidend beroepsonderwijs (LTS, LEAO, LHNO, VMBO)
- ☐ Middelbaar algemeen voortgezet onderwijs (MAVO, (M)ULO, MBO-kort, VMBO-t)
- ☐ Middelbaar beroepsonderwijs en beroepsbegeleidend onderwijs (MBO-lang, MTS, MEAO, BOL, BBL, INAS)
- ☐ Hoger algemeen en voorbereidend wetenschappelijk onderwijs (HAVO, VWO, Atheneum, Gymnasium, HBS, MMS)
- ☐ Hoger beroepsonderwijs (HBO, HTS, HEAO, HBO-V, kandidaats wetenschappelijk onderwijs)
- ☐ Wetenschappelijk onderwijs (universiteit)
- ☐ Anders, namelijk:____

> Categories were condensed to:
> 1=Low to medium
> 2= High

7.3 Wat is het netto inkomen van uw huishouden
(hiermee wordt het netto inkomen van uw volledige huishouden bedoeld en niet alleen uw inkomen)?

- ☐ €0–1200 per maand
- ☐ €1200–1800 per maand
- ☐ €1800–2600 per maand
- ☐ €2600–4000 per maand
- ☐ >€4000 per maand

> First two categories were condensed to:
> 1= €0–1800 per maand
> 2= €1800–2600 per maand
> 3=€2600–4000 per maand
> 4= >€4000 per maand
> 2,3,4 remain the same

7.4 Zijn er mensen buiten uw huishouden die geheel of gedeeltelijk van dit inkomen leven? (Denk aan kinderen op de universiteit, alimentatie voor een ex-partner, ouders in India). Als u een student bent die van een sociale uitkering leeft, is dit niet op u van toepassing. Gaat u alstublieft verder met de volgende vraag. _____________(vul aantal personen in)

7.5 Heeft u in het afgelopen jaar moeilijkheden gehad om rond te komen met uw inkomen?

- ☐ Nee, geen moeilijkheden
- ☐ Nee, geen moeilijkheden, maar ik moet wel opletten wat ik uitgeef
- ☐ Ja, enigszins moeilijkheden
- ☐ Ja, erge moeilijkheden

This question was not used for analysis

7.6 Welke situatie is het meest op u van toepassing?

- ☐ Ik heb betaald werk voor 32 uur of meer per week
- ☐ Ik heb betaald werk, tussen de 20 en 32 uur per week
- ☐ Ik heb betaald werk, tussen de 12 en 20 uur per week
- ☐ Ik heb betaald werk voor minder dan 12 uur per week
- ☐ Ik ben (vervroegd) met pensioen (AOW, VUT, FPU)
- ☐ Ik ben werkloos/werkzoekend (geregistreerd bij het arbeidsbureau)
- ☐ Ik ben arbeidsongeschikt (WAO, AAW, WAZ, WAJONG, WIA)
- ☐ Ik heb een bijstandsuitkering
- ☐ Ik ben fulltime huisvrouw/huisman
- ☐ Ik volg onderwijs/ik studeer
- ☐ Anders, namelijk ____

Categories were condensed to:
1=Unable to work/ unemployed
2= Paid job

Dit is het einde van de vragenlijst. Bedankt voor uw tijd en geduld bij het invullen van deze vragenlijst!

Als u opmerkingen heeft, kunt u deze onderaan deze pagina schrijven. Uw suggesties en feedback zijn belangrijk voor ons onderzoek.

Opmerkingen

VOUW NA HET INVULLEN VAN DE VRAGENLIJST DEZE IN DE BIJGELEVERDE RETOURENVELOPPE EN DOE DEZE OP DE POST.
