# Supplementary material for: Predictors of oral healthcare utilization and satisfaction among Indian migrants and the host population in the Netherlands
Source: BMC Oral Health. 2024 Oct 15;24:1228. doi: 10.1186/s12903-024-04988-y (PMC11481359; doi:10.1186/s12903-024-04988-y)
Supplement: Supplementary file 2 — Supplementary Material 2 [file 12903_2024_4988_MOESM2_ESM.pdf]

IDENTIFICATION CODE

ORAL HEALTH STATUS, BEHAVIORS AND CARE UTILIZATION AMONG INDIAN MIGRANTS LIVING IN THE NETHERLANDS COMPARED TO THE DUTCH POPULATION

QUESTIONNAIRE

CONSENT FORM

Oral health status, behaviors and care utilization among Indian migrants living in The Netherlands compared to the Dutch population

- I have read the information letter. I have been given the opportunity to ask questions. My questions have been answered. I have been given enough time to decide whether I want to participate.
- I know that participation is entirely voluntary and that I can decide at any time not to participate anymore. I do not have to give a reason for that.
- I consent to participate in this study and fill the questionnaire as a part of this study.
- I also give my permission to the researcher to use my information for the purpose stated in the information letter. I consent to save my information for 5 years after this study.
- As explained, all my personal information will be treated confidentially and will be included anonymously in this study.

☐ I give my permission to participate in the study

☐ I do not give my permission to participate in the study

Signature ___________________________

Date _______________________________

VU VRIJE UNIVERSITEIT AMSTERDAM

UNIVERSITEIT VAN AMSTERDAM

ACADEMISCH CENTRUM TANDHEELKUNDE AMSTERDAM
ACTA
GUSTAV MAHLERLAAN 3004
1081 LA AMSTERDAM

QUESTIONNARE – ORAL HEALTH AMONG INDIAN IMMIGRANTS IN THE NETHERLANDS

You should complete this questionnaire yourself.

- Please take your time and read the questions carefully. If you aren't able to answer the questions by yourself, you can ask someone to help you. But if you do, it's important that the answers you give are your own. If there's no one you can ask for help or if you feel the need to get help in understanding or reading the questions, please feel free to contact the investigator (Phone: 0626870875).
- Also, if you aren't sure how to answer a question, try to give the one closest to your own situation. It's important to answer all of the questions. After some answers, you are told to go to a question further on in the questionnaire, then you can skip the questions in between.
- You don't need to fill in the whole questionnaire all at once. You can fill in part of it and finish the rest later.
- It will take approximately 30-40 minutes for you to fill this questionnaire. All these questions are important as they will help us gather data not only on your oral/ dental health, but will also give us an insight into the day-to-day activities/ problems related to your health and oral health, while you live in The Netherlands.
- Although there are no 'right' or 'wrong' answers, it's important your answers are honest.
- This questionnaire is available online via https://tiny.cc/koa5tz in English, Hindi and Dutch. If you would like to fill this questionnaire online or in any other language (Dutch or Hindi), please copy this link on your computer and you will be directed to the online questionnaire.
- You can also use this QR code and scan the code. This will also direct you to the digital questionnaire
- If you are filling this paper version of the questionnaire, then after completing the questionnaire, kindly fold this entire folder and place it in the return envelope provided and post it. You do not need to stamp the return envelope.

SECTION 1: GENERAL INFORMATION

1.1 What is your gender?

- ☐ Male
- ☐ Female
- ☐ Others

1.2 How old are you? ____________ *years*

1.3 What is your present marital status?

- ☐ Married /Registered partnership
- ☐ Cohabiting (living together)
- ☐ Unmarried (never married)
- ☐ Divorced or Separated
- ☐ Widow / Widower

1.4 Including yourself, how many people are there in your household? If you live alone, your answer is ‘1’. _____________

1.5 What is your nationality (As mentioned in your passport)? ______________________

1.6 What is your postal code (Please enter the first four digits only) _________________

1.7 Do you plan to settle in the Netherlands?

- ☐ Yes
- ☐ No
- ☐ I am not sure yet

1.8 To settle in The Netherlands you need to apply for permanent residence. Have you applied for permanent residence?

- ☐ No
- ☐ Yes, I have applied / will apply for permanent residence
- ☐ Yes, I already have the permanent residence

SECTION 2: COUNTRY OF BIRTH AND CULTURE

2.1 What is your country of birth?

- ☐ India
- ☐ The Netherlands
- ☐ Other country, please specify ____

2.2 In which year did you come to live in the Netherlands? ______________________

2.3 Which year did you leave your country of birth? ________________ . If you do not remember the exact year, kindly make an estimation.

2.4 What is the most important reason for your moving to the Netherlands? *(You can choose more than one option).*

- ☐ I came with my parents
- ☐ I came to work
- ☐ I came to study
- ☐ I came to join my parents who were already living in the Netherlands
- ☐ I came to join my partner/ husband/ wife
- ☐ I came to marry someone who is already living in the Netherlands
- ☐ For the social security system in the Netherlands
- ☐ Because of financial reasons in my home country
- ☐ Any other reason, please specify ______________________________________

2.5 Which region of India are you from? Specify your state and city name.

____

2.6 There are various religions in India, which one do you belong to?

- ☐ None
- ☐ Hindu
- ☐ Muslim
- ☐ Sikh
- ☐ Christian
- ☐ Jain
- ☐ Other, please specify ____

2.7 How connected do you feel with The Netherlands?

- ☐ I feel an extremely close connection.
- ☐ I feel a very close connection
- ☐ I feel a moderately close connection
- ☐ I feel a weak connection
- ☐ I do not feel a connection at all

2.8 How often do you feel like an outsider in The Netherlands?

- ☐ Never
- ☐ Rarely
- ☐ Sometimes
- ☐ Often
- ☐ Always

2.9 Before the COVID lockdown, how often did you eat dinner with Dutch people who are *not* part of your family?

- ☐ Never
- ☐ Once a year
- ☐ Once a month
- ☐ Once a week
- ☐ Almost every day

2.10 Communicating in Dutch has many components, like reading, listening, writing, and speaking skills. Please evaluate your own skills in Dutch language. How well can you do the following when reading, speaking, writing, or listening to Dutch? Please mark one answer for each row.

		Not well at all	Not well	Moderately well	Well	Very well
a	I can read and understand the main points in simple newspaper articles on familiar subjects.	☐	☐	☐	☐	☐
b	In a conversation, I can speak about familiar topics and express personal opinions.	☐	☐	☐	☐	☐

2.11 In The Netherlands, how difficult or easy would it be for you to do each of the following?

		Very difficult	Somewhat difficult	Neither difficult nor easy	Somewhat easy	Very easy
a	See a doctor	☐	☐	☐	☐	☐
b	See a dentist	☐	☐	☐	☐	☐
c	Search for jobs (find job vacancies)	☐	☐	☐	☐	☐
d	Get help with legal problems	☐	☐	☐	☐	☐

2.12 Please think about the Dutch people in your address book or your phone contacts. With how many of them did you have a conversation - either by phone, messenger chat, or text exchange - *in the last 4 weeks*?

- ☐ 0
- ☐ 1 to 2
- ☐ 3 to 6
- ☐ 7 to 14
- ☐ 15 or more

2.13 How well do you understand the important political issues facing The Netherlands?

- ☐ Very well
- ☐ Well
- ☐ Moderately well
- ☐ Not well
- ☐ Not well at all

2.14 In the last 12 months, how often did you typically discuss major political issues facing The Netherlands with others?

- ☐ Never
- ☐ Once a year
- ☐ Once a month
- ☐ Once a week
- ☐ Almost every day

2.15 In your daily life, how often have the following events happened to you because of your background?

		Never	Hardly ever	Not too often	Fairly often	Very often
a	You are treated with less politeness	☐	☐	☐	☐	☐
b	You are treated with less respect	☐	☐	☐	☐	☐
c	You receive poorer service than other people (in restaurants or shops)	☐	☐	☐	☐	☐
d	People act like they think you are not smart	☐	☐	☐	☐	☐
e	People act like they are afraid of you	☐	☐	☐	☐	☐
f	People act like they think you are dishonest	☐	☐	☐	☐	☐
g	People act like they are better than you are	☐	☐	☐	☐	☐
h	You are threatened or harassed	☐	☐	☐	☐	☐

SECTION 3: LIFESTYLE AND HABITS

3.1 Do you smoke?

- ☐ Yes, I smoke
- ☐ No, I never smoked (*If you tick this answer, then kindly proceed to question no. 3.3*)
- ☐ No, but I smoked in the past

3.2 When did you start smoking?

- ☐ Before moving to The Netherlands
- ☐ After moving to The Netherlands

3.3 How often do you chew tobacco?

- ☐ Never
- ☐ Less than once a month
- ☐ Once a month
- ☐ 2 to 4 times a month
- ☐ 2 to 4 times a week
- ☐ 5 to 6 times a week
- ☐ Daily

3.4 How often do you have drinks that contain alcohol?

- ☐ Never *If you tick this answer, then kindly proceed to question no. 3.6*
- ☐ Less than once a month
- ☐ Once a month
- ☐ 2 to 4 times a month
- ☐ 2 to 4 times a week
- ☐ 5 to 6 times a week
- ☐ Daily

3.5 When did you start drinking?

- ☐ Before moving to The Netherlands
- ☐ After moving to The Netherlands

3.6 How often do you consume sugary Indian-Sweets (methai)?

- ☐ Rarely/ Never
- ☐ Less than once a month
- ☐ Once a month
- ☐ 2 to 4 times a month
- ☐ 2 to 4 times a week
- ☐ 5 to 6 times a week
- ☐ Daily

3.7 How often do you consume sweets like cakes and chocolates?

- ☐ Rarely/ Never
- ☐ Less than once a month
- ☐ Once a month
- ☐ 2 to 4 times a month
- ☐ 2 to 4 times a week
- ☐ 5 to 6 times a week
- ☐ Daily

3.8 How often do you consume fizzy drinks?

- ☐ Rarely/ Never
- ☐ Less than once a month
- ☐ Once a month
- ☐ 2 to 4 times a month
- ☐ 2 to 4 times a week
- ☐ 5 to 6 times a week
- ☐ Daily

3.9 Do you add sugar to hot drinks?

- ☐ Yes, always
- ☐ Yes, sometimes
- ☐ No, never

3.10 Do you think your sugar consumption has changed since you migrated to The Netherlands?

- ☐ Yes, it has increased
- ☐ Yes, it has decreased
- ☐ No, it stayed the same
- ☐ I don’t know

The following questions are about the habits you have towards cleaning your mouth.

3.11 What do you use to clean your teeth? (*You can choose more than one option*)

- ☐ Manual tooth brush
- ☐ Electric tooth brush
- ☐ Tooth paste
- ☐ Tooth powder
- ☐ Chewing stick
- ☐ Homemade powder. Please specify the ingredients________________________
- ☐ Any other method, please specify _____________________________________

3.12 How often do you use the following? *Please provide answer for each item.*

		Daily	Several times a week	Once a week	Every 2 weeks	Once a month	Never
a	Tooth picks	☐	☐	☐	☐	☐	☐
b	Dental floss	☐	☐	☐	☐	☐	☐
c	Interdental brushes	☐	☐	☐	☐	☐	☐
d	Mouth wash	☐	☐	☐	☐	☐	☐
e	Any other, namely ____________	☐	☐	☐	☐	☐	☐

3.13 Do you use toothpaste with fluoride in it?

- ☐ Yes
- ☐ No
- ☐ I don’t know

3.14 Do you use any alternative dental care method (traditional methods or home remedies)? If yes, what will you do for the following ailments? *Otherwise go to question 3.15),*

- ☐ For toothache ______________________________
- ☐ For bleeding gums ______________________________
- ☐ For bad breath ______________________________
- ☐ For tooth loss ______________________________
- ☐ For sharp or irritating tooth ______________________________
- ☐ For mouth ulcers ______________________________

3.15 How confident are you, that you clean your teeth in the following situations?

		Completely confident	Fairly confident	Not so confident	Not confident at all
a	When you are tired in the evening	☐	☐	☐	☐
b	When you are not going to the dentist in the near future	☐	☐	☐	☐
c	When you are on holiday	☐	☐	☐	☐
d	When you have a lot of work	☐	☐	☐	☐
e	When you have a headache or feel ill	☐	☐	☐	☐

3.16 How will you rate the following statements?

		Strongly disagree	Dis agree	Agree	Strongly agree
a	I will have tooth decay whatever I do, because I cannot change the structure of my teeth	☐	☐	☐	☐
b	I can prevent tooth decay and gum diseases if I brush my teeth regularly	☐	☐	☐	☐
c	I can maintain my oral health only with information from my dentist	☐	☐	☐	☐
d	I think that whatever caused the deterioration of my oral health is due to bad luck	☐	☐	☐	☐
e	I immediately go to my dentist whenever I feel that something is wrong with my oral health	☐	☐	☐	☐
f	The warnings of my family have an important influence on my oral health	☐	☐	☐	☐
g	My dental diseases will improve rapidly if I am lucky enough	☐	☐	☐	☐
h	I am aware of what to do to maintain my oral health	☐	☐	☐	☐
i	The warnings of my friends and people in my close environment have an important influence on my oral health	☐	☐	☐	☐
j	If my oral health condition worsens, it's a matter of fate	☐	☐	☐	☐
k	I am directly responsible for my oral health	☐	☐	☐	☐
l	My oral health depends on dentists	☐	☐	☐	☐
m	When my oral health worsens, I leave everything to its course and say, “I will bear whatever I have to suffer”	☐	☐	☐	☐
n	I blame myself if I have tooth decay	☐	☐	☐	☐
o	Dentists maintain my oral health	☐	☐	☐	☐
p	My gums will be diseased and will bleed whatever I do, because I cannot change their structure	☐	☐	☐	☐

		Strongly disagree	Disagree	Agree	Strongly agree
q	The only factor that maintains my oral health is my care for my oral health	☐	☐	☐	☐
r	I owe the avoidance of tooth decay and gum diseases and the improvement in my oral health to my dentists	☐	☐	☐	☐
s	My teeth will be healthy in the long term if I care for them	☐	☐	☐	☐
t	I will prevent tooth decay and gum diseases if I care for my oral health properly	☐	☐	☐	☐
u	I do only what is told by the dentist	☐	☐	☐	☐
v	I know that I have neglected caring for my teeth when I have problems with my oral health	☐	☐	☐	☐
w	Luck plays a big part in determining how soon I will recover from tooth decay and gum disease	☐	☐	☐	☐
x	I take care to maintain and improve my oral health	☐	☐	☐	☐
y	It is possible to have healthy teeth in the long term if I do what should be done	☐	☐	☐	☐
z	The only way to maintain my oral health is to comply with the dentists' recommendations	☐	☐	☐	☐

SECTION 4: ORAL HEALTH STATUS

4.1 How would you rate the health of your mouth and teeth?

- ☐ Very good
- ☐ Good
- ☐ Fair
- ☐ Poor
- ☐ Very poor

4.2 Adults can have up to 32 natural teeth, including wisdom teeth, but over time people lose some of them.

- Please count the number of natural teeth in your upper jaw ______ [fill in a number between 0 and 16]
- Please count the number of natural teeth in your lower jaw ______ [fill in a number between 0 and 16]

4.3 Do you have a removable denture in your upper jaw?

☐ Yes, full denture

☐ Yes, partial denture

☐ No

4.4 Do you have a removable denture in your lower jaw?

☐ Yes, full denture

☐ Yes, partial denture

☐ No, I don't have any artificial tooth in my lower jaw

4.5 Has your dentist/ hygienist ever told you that you have gum disease (periodontal disease)?

☐ Yes

☐ No

☐ I don't know/remember

4.6 Do you think your teeth are loose (mobile) in your mouth?

☐ No

☐ Slightly loose (mobile)

☐ Highly loose (mobile)

4.7 What will you decide if you have dental pain due to persistent swelling?

- ☐ Try and control the pain with medicines and wait for the pain to go away
- ☐ Get dental treatment immediately
- ☐ Discuss with the family first and decide based on what they say
- ☐ Decide to wait for the treatment till you visit your native country when going for vacations
- ☐ Any other, please specify ____

4.8 During the last 3 months have you had any of the following problems in your mouth?

		Never	Less than once a month	About 1-3 times a month	About 1-2 times a week	About 3-4 times a week	Almost every day / Daily
**a**	Toothache	☐	☐	☐	☐	☐	☐
**b**	Bleeding or swollen gums	☐	☐	☐	☐	☐	☐

4.9 We would like to know about the severity of any difficulties or problems caused by your mouth, teeth or dentures. Using the scale from 0 to 5, where 0 is no effect and 5 is a very severe effect, can you tell us what effect the following difficulties and problems have had on your daily life in the past 12 months?

		No effect →					Very severe effect
a	Difficulty eating	0 ☐	1 ☐	2 ☐	3 ☐	4 ☐	5 ☐
b	Difficulty speaking	0 ☐	1 ☐	2 ☐	3 ☐	4 ☐	5 ☐
c	Difficulty cleaning your teeth or dentures	0 ☐	1 ☐	2 ☐	3 ☐	4 ☐	5 ☐
d	Difficulty going out, for example to the shops or visiting someone	0 ☐	1 ☐	2 ☐	3 ☐	4 ☐	5 ☐
e	Difficulty relaxing (including sleeping)	0 ☐	1 ☐	2 ☐	3 ☐	4 ☐	5 ☐
f	Problems smiling, laughing and showing teeth without embarrassment	0 ☐	1 ☐	2 ☐	3 ☐	4 ☐	5 ☐
g	Difficulty carrying out your major work or role	0 ☐	1 ☐	2 ☐	3 ☐	4 ☐	5 ☐
h	Problems with emotional instability, for example becoming more easily upset than usual	0 ☐	1 ☐	2 ☐	3 ☐	4 ☐	5 ☐
i	Problems enjoying the contact of other people, such as relatives, friends or neighbours	0 ☐	1 ☐	2 ☐	3 ☐	4 ☐	5 ☐

SECTION 5: ORAL HEALTH SERVICE UTILIZATION

5.1 Where do you usually visit the dentist?

- ☐ I only visit the dentist in India, when I go back
- ☐ I only visit the dentist in The Netherlands
- ☐ I visit the dentist both in India and in The Netherlands
- ☐ I have never visited a dentist. *If you tick this option, kindly proceed to question no. 5.9*

5.2 Before the COVID lockdown, have you visited a dentist?

- ☐ Yes
- ☐ No
- ☐ I don't remember

5.3 Before the COVID lockdown, have you visited a dental hygienist?

- ☐ Yes
- ☐ No
- ☐ I don't remember

5.4 What is your usual reason for visiting the dentist? *(You can choose more than one option)*

- ☐ Consultation or Advice
- ☐ Pain or trouble with teeth, gums or mouth
- ☐ Treatment or follow- up treatment
- ☐ Routine check-up
- ☐ I don't remember
- ☐ Any other reason, please specify ____

5.5 How far is the dental clinic from your residence in The Netherlands?

- ☐ In the neighbourhood
- ☐ Less than 30 minutes away
- ☐ Between 30 minutes and one hour away
- ☐ More than one hour away
- ☐ I don't know

5.6 Before the COVID lockdown, have you received any of the following treatments?

		Yes	No	I do not remember
**a**	Treatment for gum disease (periodontitis)	☐	☐	☐
**b**	New filling	☐	☐	☐
**c**	Root canal treatment	☐	☐	☐
**d**	Tooth removed	☐	☐	☐
**e**	New artificial tooth (crown or bridge or implant)	☐	☐	☐
**f**	Teeth cleaning	☐	☐	☐
**g**	Treatment done purely to improve the appearance of your teeth (restoration in front teeth, teeth whitening, orthodontic treatment)	☐	☐	☐

5.7 What do you look for when searching for a dentist? (*You can choose more than one option*)

- ☐ Availability of an interpreter
- ☐ I should be able to discuss my dental problems clearly with the dentist
- ☐ Sometimes, I should be allowed to make an appointments at my own convenience
- ☐ My dentist should be able to give me priority in case I have any dental emergency
- ☐ Whether the dental staff is friendly and welcoming
- ☐ Dental clinic that looks clean and hygienic
- ☐ Dental clinic closer to my house or work place
- ☐ Dental clinic based on recommendations of colleagues or friends or other family members
- ☐ I compare the prices of the dental treatment and then choose my dentist
- ☐ Whether there are more people from my community visiting the dentist
- ☐ Whether my dentist understands my cultural background
- ☐ Any other, please specify ____

5.8 How satisfied are you with your dentist?

- ☐ Completely satisfied
- ☐ Reasonably satisfied
- ☐ Neutral
- ☐ Reasonably unsatisfied
- ☐ Completely unsatisfied

5.9 If you have not visited a dentist, what are your reasons (*You can choose more than one option*). If you have answered questions 5.2 to 5.8, kindly proceed to *question 5.10*

- ☐ Nothing is wrong with my teeth
- ☐ Language problem
- ☐ Dental fear or Anxiety
- ☐ Lack of confidence in the dental clinic
- ☐ Problem goes away with time
- ☐ Dental visits cost a lot
- ☐ Others, please specify ____

5.10 Do you have dental insurance?

- ☐ Yes
- ☐ No
- ☐ I do not know

SECTION-6: GENERAL HEALTH

6.1 How will you **rate** your overall **general health**?

- ☐ Very good
- ☐ Good
- ☐ Fair
- ☐ Poor
- ☐ Very poor

6.2 Did you have any of the following health problems? *(You can choose more than one option in each row)*

		Yes, before moving to the NL	Yes, after moving to the NL	No
a	Diabetes	☐	☐	☐
b	Blood pressure	☐	☐	☐
c	Serious heart condition (for example, heart failure or severe chest pain)	☐	☐	☐
d	Cancer	☐	☐	☐
e	Others	☐	☐	☐

If you choose 'others' then please name the health problem here ____________________

6.3 Do you use traditional medicines like Ayurveda, naturopathic, etc for your health problems?

- ☐ Yes
- ☐ No
- ☐ I don't know

The next 5 questions only need to be filled in <u>if you are diagnosed with diabetes.</u>

<u>Otherwise,</u> *<u>you can proceed to question 7.1</u>*

6.4 Have you had a test for high blood sugar within the past three years?

- ☐ Yes
- ☐ No

6.5 How old were you when you were first diagnosed with diabetes? If you aren't sure, please try to estimate this. __________ years old

6.6 Has someone in your immediate family (your parents, brothers, sisters, or children) been diagnosed with diabetes?

- ☐ Yes
- ☐ No
- ☐ I don't know

6.7 Has a doctor or specialist treated you for diabetes <u>in the past 12 months</u>?

- ☐ Yes
- ☐ No

SECTION 7: SOCIAL SUPPORT STRUCTURE

7.1 What kind of support do you usually get?

		All the time	Most of the time	Often	Some time	Never	Not applicabl e
a	Someone who gives you love and affection	☐	☐	☐	☐	☐	☐
b	Someone you can have a nice day with	☐	☐	☐	☐	☐	☐
c	Someone who you can trust to talk about yourself or your problems	☐	☐	☐	☐	☐	☐
d	Someone you meet for relaxation	☐	☐	☐	☐	☐	☐
e	Someone who will cook for you if you can't handle it	☐	☐	☐	☐	☐	☐
f	Someone who can help you with your daily work if you are ill	☐	☐	☐	☐	☐	☐
g	Someone with whom you share your most confidential concerns and fears	☐	☐	☐	☐	☐	☐
h	Someone to whom you can go for suggestions on how to deal with a personal problem	☐	☐	☐	☐	☐	☐
i	Someone you love and who makes you feel worthy	☐	☐	☐	☐	☐	☐

SECTION 8: EDUCATION AND EMPLOYMENT STATUS

8.1 What is the highest level of education you have completed in the Netherlands? (*This is the highest level of education you completed and for which you received a diploma or a certificate of proficiency*).

- ☐ None (have had no formal education, did not finish education)
- ☐ Primary education (primary school, special primary education)
- ☐ Lower or preparatory vocational education (LTS, LEAO, LHNO, VMBO)
- ☐ Junior general secondary education (MAVO, (M)ULO, MBO-kort, VMBO-t)
- ☐ Upper secondary vocational education and apprenticeship training (MBO-lang, MTS, MEAO, BOL, BBL, INAS)
- ☐ Senior general secondary education and pre-university education (HAVO, VWO, atheneum, gymnasium, HBS, MMS)
- ☐ Higher professional education (HBO, HTS, HEAO, HBO-V, kandidaats wetenschappelijk onderwijs)
- ☐ University
- ☐ Other, namely ______________________________

8.2 What is the highest level of education you completed in India, for which you received a diploma, (*This is the highest level of education you completed and for which you received a diploma or a certificate of proficiency*).

- ☐ None (have had no formal education, did not finish education)
- ☐ Primary school literate (Up to class VIII)
- ☐ Middle school certificate (Class VIII and class IX pass)
- ☐ High school certificate (Class X pass)
- ☐ Higher secondary certificate (Class XII pass)
- ☐ Graduate degree (Any graduation degree such as B.A, B.Sc, B.Ed)
- ☐ Post graduate or professional degree (M.A, M.Sc, Ph.D, M.Ed, M.B.B.S, B.E, B.Arch)
- ☐ Any vocational training, please specify______________________________
- ☐ Other, namely ______________________________

8.3 What is the net income of your household? (*This relates to the income of the whole household, not just your own*).

- ☐ €0–1200/month
- ☐ €1200–1800/month
- ☐ €1800–2600/month
- ☐ €2600–4000/month
- ☐ >€4000/month

8.4 Are there people outside your household who live entirely or partially from this income? (*Think of children away at university, alimony for an ex-partner, parents back in India*). If you are a student living on social benefits, this is not applicable to you. Kindly proceed to the next question.
________________________________ (please enter the number of people).

8.5 During the past year, did you have difficulties managing your household income?

- ☐ No
- ☐ No, but I have to watch what I spend
- ☐ Yes, some difficulties
- ☐ Yes, a lot of difficulties

8.6 Which situation most applies to you?

- ☐ I have a paid job, and work 32 or more hours a week
- ☐ I have a paid job, and work between 20 and 32 hours a week
- ☐ I have a paid job, and work between 12 and 20 hours a week
- ☐ I have a paid job, and work less than 12 hours a week
- ☐ I am retired (AOW, VUT, FPU)
- ☐ I am unemployed and looking for work (registered with the job center)
- ☐ I am unable to work (WAO, AAW, WAZ, WAJONG, WIA)
- ☐ I get social benefits (‘bijstandsuitkering’)
- ☐ I am a full-time homemaker (male or female)
- ☐ I am a student (including those who are presently on vacation)
- ☐ Other, namely: ____

After filling this questionnaire, you might be interested to talk more about your experiences. If you want to discuss this further and provide better oral health care to your family and children, we need to come together and make our voices heard!

For this, we would like to invite you for a group discussion to discuss your views, opinions and experiences regarding your oral health and dental visits in the Netherlands.

In case you are interested, we would request you to kindly provide us with your email address and / or your contact details so that we can contact you.

- Email address ____
- Phone number (optional)_______________________________

Pleases remember, we will use this personal information for inviting you for group discussions only, following which we will delete this information completely.

In case you need to contact the researcher for further queries or questions or clarifications on the given questions, kindly contact us on the following email: a.pabbla@acta.nl or phone number 0626870875.

If you have any comments, you can write them at the bottom of this page. Your suggestions and feedback is important to our research.

Comments

We look forward to your positive responses

This is the end of the questionnaire. Thank you for your time and patience in filling out this questionnaire

AFTER FILLING IN THE COMPLETE QUESTIONNAIRE, KINDLY FOLD THIS ENTIRE FOLDER AND PLACE IT IN THE RETURN ENVELOPE PROVIDED AND POST IT.
